# A Clinicohistopathological Characterization of Oral Lichen Planus: A Cross-Sectional Study

**DOI:** 10.7759/cureus.30568

**Published:** 2022-10-21

**Authors:** Aswathy K. Vijayan, Arvind Muthukrishnan

**Affiliations:** 1 Oral and Maxillofacial Radiology, PMS College of Dental Science and Research, Trivandrum, IND; 2 Oral Medicine & Radiology, Saveetha Dental College & Hospital, Chennai, IND

**Keywords:** plasma cells, oral lichen planus, koilocytosis, hydropic degeneration, epithelial dysplasia

## Abstract

Objective: Based on the modified diagnostic criteria for oral lichen planus (OLP) proposed by Van der Meij and Van der Waal, the objective of the current investigation was to demonstrate a clinicohistopathological association in the diagnosis of OLP.

Materials and methods: Data were retrieved from 250 individuals who visited the Department of Oral Medicine and Radiology and were diagnosed with OLP between September 2018 and December 2021. Upon completion of the histopathological analysis, the precise diagnosis of OLP was made. Repeat biopsies were performed in the cases suspecting malignant transformation during the follow-up phase. The data were analyzed using SPSS software. The Fisher's exact test and chi-square test of association were used to establish the significant differences between the variables at a 5% significance level.

Results: Of the 250 patients, 48% and 52% were males and females, respectively. The two clinical manifestations observed were reticular (n=145, 58%) and erosive types (n=105, 42%). The most frequently impacted locations were the buccal mucosa (n=150, 60%) and labial mucosa (n=100, 40%). Fourteen individuals (two with reticular form and 12 with erosive form) later during follow-up showed dysplasia, with moderate (n=2) to mild (n=12) dysplastic alterations. Koilocytes were reported in 84 cases (34%), which included 35 (24%) reticular cases and 49 (47%) erosive lesions. The histopathological features such as acanthosis, epithelial atrophy, hyperkeratosis, presence of neutrophils, koilocytes, and epithelial dysplasia were shown to be statistically significant between the clinical forms (p<0.001).

Conclusion: The results of the current study highlight the concordance of histopathological and clinical diagnoses, especially for early definitive diagnosis of OLP. More research studies are warranted to validate the trend of epithelial dysplasia in OLP associated with human papillomavirus (HPV) and to explore the course of the lesions that might be affected by this trait.

## Introduction

Oral lichen planus (OLP) is a frequent condition affecting the skin and mucous membrane of the oral cavity which is of unknown cause [1]. As its pathogenesis is not well understood, the clinicopathological diagnostic criteria for OLP have been continuously altered. The inflammatory component in OLP is intense and is the result of a response to the antigens produced by the basal keratinocytes. The basal layer of the epithelium degenerates as a result of the inflammatory process of these self-antigens. The only area with discernible inflammation is the band-like subepithelial connective tissue. CD8+ T lymphocytes, which are present at the epithelial layer, are largely responsible for the manifestation of inflammation [2-4].

The pathophysiology of OLP may be immune-mediated by both antigen-specific and non-specific pathways. Basal keratinocytes release the antigen in OLP, and CD8+ cytotoxic T lymphocytes destroy keratinocytes specifically in response to the antigens. Activation of matrix metalloproteinase and degranulation of mast cells are examples of non-specific pathways in OLP. T-cell aggregation in the superficial lamina propria, rupture of the basement membrane, T-cell migration within the epithelium, and apoptosis of keratinocytes are the possible outcomes of the interaction of these pathways. Furthermore, inadequate immunosuppression, which is mediated by transforming growth factor-beta, may contribute to the chronic characteristic feature of this condition [5].

One of the clinical features is the existence of bilateral, symmetrical white reticular lesions. The lesions might be atrophic, erosive, bullous, or plaque-like variants and coexist with reticular lesions. Both conditions must be met for an OLP to be considered typical. Clinically compatible with lichen planus is a term used to describe lesions that resemble an OLP but do not match the aforementioned requirements [3,6]. The reticular variant is more common and is marked by definite erythematous borders around Wickham's striae, which are lacy white streaks. Although the erosive type is less frequent than the reticular form, it is more pertinent for patients since lesions are frequently bothersome [5]. Mild discomfort to excruciatingly painful bouts that make it challenging to chew is indeed a possible symptom. Clinical signs of erosive lichen planus include atrophic and erythematous patches that are frequently encircled by thin striae that radiate outward. Bullous lichen planus, a very uncommon variant of the disease, can develop in some situations where the epithelium may detach if erosion is severe. Desquamative gingivitis occurs occasionally where atrophy and ulceration are limited to the gingival tissue [5]. They are frequently categorized into two groups in an effort to enhance clinicopathological association: those with solely reticular lesions and those with atrophic/erosive lesions with or without accompanying reticular lesions [7].

OLP has been related to several generalized medical conditions, including medications for diabetes, hypertension, and certain immunological conditions. The recent findings that human papillomavirus (HPV) is identified in a substantial portion of oral lesions reinforce the notion that the condition may have viral correlates. There have already been reported OLP biopsies with HPV-11, 16, and HPV-16-related viruses. There still is not sufficient evidence to make this correlation. Ninety percent of HPV-associated head and neck cancers and 50% of oropharyngeal head and neck squamous cell carcinomas have been found to harbor HPV type-16 [1]. Subclinical and/or latent infections of the oral cavity are associated with an HPV prevalence rate of 22% to 60% in the normal mucosa [8].

In India, the prevalence of HPV varies greatly by region, with 27% of oral cancer cases from North India testing positive for HPV-16, compared to 25% to 31% in western India. Uncertainty nonetheless exists regarding the precise mode of entry of HPV into cells. It is widely acknowledged that HPV binder proteins allow the virus to enter the epithelium and transfer deoxyribonucleic acid (DNA) to the nucleus. The virus infects the new host when it enters through micro-wounds. Koilocytosis is the most typical cytomorphologic alteration and is regarded by pathologists as a key histopathologic characteristic for the detection of HPV infection. Despite the fact that koilocytosis is a significant morphological indicator of HPV infection, occasionally, koilocyte-like abnormalities result from fixation, processing, or procedural errors during biopsy [8]. The overrepresentation of OLP by the World Health Organization (WHO) 1978 criteria is addressed by the Van der Meij criterion. Consequently, to objectively confirm the final diagnosis, a number of exclusion criteria were included such as appropriate bilateral clinical manifestations and the occurrence of at least a mild reticular feature. Histopathologically, the appearance of a subepithelial inflammatory zone with basal cell degeneration and a predominance of T cells was regarded as a typical diagnostic of OLP. The clinical and/or histological assessment was regarded as not typical but only compatible in the lack of any one of these traits and was thus excluded from the diagnosis of OLP [2].

The phase of the disease during the biopsy, as well as any recent treatments for OLP, may have an impact on its histological characteristics. There are divergent views on the distinctions based on the reticular or erosive variant of OLP and the site of the biopsy in the oral cavity. It is primarily distinguished by a subepithelial band-like lymphocytic infiltration and the occurrence of intraepithelial lymphocytes [7]. On both clinical and histological levels, lichen planus can be misinterpreted for other lichenoid disorders like nonspecific lichenoid responses, atypical lichenoid stomatitis, graft-versus-host reactions, drug reactions, lupus erythematosus, erythema multiforme, and oral lichenoid dysplasia. A number of clinical and histological characteristics specific to lichen planus that the lichenoid response does not fully satisfy, as well as the absence of the distinctive trait and distribution of OLP, distinguish OLP from lichenoid reactions. However, there are limited data on the association between the histopathological and clinical diagnosis of OLP, particularly in developing nations like India [3,7,9,10].

The purpose of this study is to identify the most distinctive histopathological signs of OLP and their associations with the clinical characteristics and patterns that occur most frequently in a sample of the South Kerala population.

## Materials and methods

According to the Strengthening the Reporting of Observational Studies in Epidemiology (STROBE) guidelines for observational studies, a clinicopathological study was carried out in PMS College of Dental Sciences and Research, which was approved by the Institutional Ethics Committee with ref number PMS/IEC/2018-19/40. Data were retrieved from 250 individuals who visited the Department of Oral Medicine and Radiology and were diagnosed with OLP between September 2018 and December 2021. In compliance with the Helsinki Declaration, the study was completed after obtaining written informed consent.

Upon completion of a biopsy and its following histopathological analysis, the precise diagnosis of OLP was made. Cases were eliminated from the analysis if they failed to satisfy both the clinical and the histological characteristics unique to OLP established by the Van der Meij and Van der Waal criteria [11]. The diagnosis was made clinically based on the appearance of symmetrical and bilateral lesions with a network of lace-like grayish-white lines in a reticular pattern, with or without erosive, plaque-type, atrophic, or bullous lesions. Based on histology, lesions with a clearly delineated zone of lymphoid infiltration and liquefaction degradation in the layer of basal cells without epithelial dysplasia were taken into consideration. Patients who had a history of systemic disorders, were smokers or heavy drinkers, had immune-mediated hypersensitivity reactions to tooth restorations, or were taking any medications such as oral hypoglycemics or angiotensin-converting enzyme inhibitors were also precluded.

The clinical characteristics of the disease such as age, gender, clinical form, and anatomic site of the lesions and the histological findings seen at the epithelium and connective tissue level were subsequently recorded. Stained slides and paraffin blocks were obtained for examination in all the cases. The composition and intensity of the inflammatory response, distribution of perpetual or fragmented superficial band-like inflammatory cells as well as epithelial hyperkeratosis, epithelial atrophy, and the occurrence of ulceration were assessed histopathologically. Two independent investigators reviewed the tissue samples that had been stained with hematoxylin and eosin. The guidelines established by Patil et al. were used to evaluate the cases of epithelial dysplasia that occurred during the follow-up phase [12].

The statistical evaluations were carried out using Statistical Package for the Social Sciences (SPSS) Statistics for Windows, Version 26.0 (IBM Corp., Armonk, NY, USA). The Shapiro-Wilk test was applied to ascertain whether the data follow a normal distribution. Later, using the frequencies and percentages for the categorical variables, descriptive statistics were computed. The Fisher's exact test and chi-square test of association were used to establish the significant differences between the variables at a 5% level of significance.

## Results

Of the 250 patients, with ages ranging from 15 to 74, 48% and 52% were males and females, respectively. Males and females reported initial diagnoses on average at 35.5 and 39.1 years old, respectively. The two clinical manifestations that were most frequently seen were reticular (n=145, 58%) and erosive types (n=105, 42%). The buccal mucosa (n=150, 60%) and labial mucosa (n=100, 40%) were the two most frequently impacted locations. Even though the majority of patients had numerous oral sites of involvement, they were excluded due to the stringent exclusion criteria. The histopathologic and clinical diagnoses were consistent according to Cohen's kappa, which was 0.787. Table 1 depicts the correlation between the histological findings and the clinical presentations of the various OLP categories.

**Table 1:** Association between histological and clinical findings of OLP in the study sample **Highly significant. n, number; OLP, oral lichen planus.

	Reticular (n=145)		Erosive (n=105)		
Histological findings	Yes n (%)	No n (%)	Yes n (%)	No n (%)	p-Value
Hydropic degeneration of the basal layer	145 (100)	0 (0)	105 (100)	0 (0)	1
Acanthosis	83 (57)	62 (43)	19 (18)	86 (82)	<0.0001**
Saw-tooth rete pegs	90 (62)	55 (38)	60 (57)	45 (43)	0.43
Epithelial atrophy	0 (0)	145 (100)	42 (40)	63 (60)	<0.0001**
Epithelial hyperkeratosis	104 (72)	41 (28)	55 (52)	50 (48)	<0.001**
Subepithelial lymphocytic infiltrate	145 (100)	0 (0)	105 (100)	0 (0)	1
Plasma cells in the connective tissue	45 (31)	100 (69)	44 (42)	61 (58)	0.07
Neutrophils in epithelium	0 (0)	145 (100)	60 (57)	45 (43)	<0.0001**
Koilocytes	35 (24)	110 (76)	49 (47)	56 (53)	0.0001**
Epithelial dysplasia	2 (1)	143 (99)	12 (11)	93 (89)	0.0006**

Histological features at the time of diagnosis

**Figure 1:**
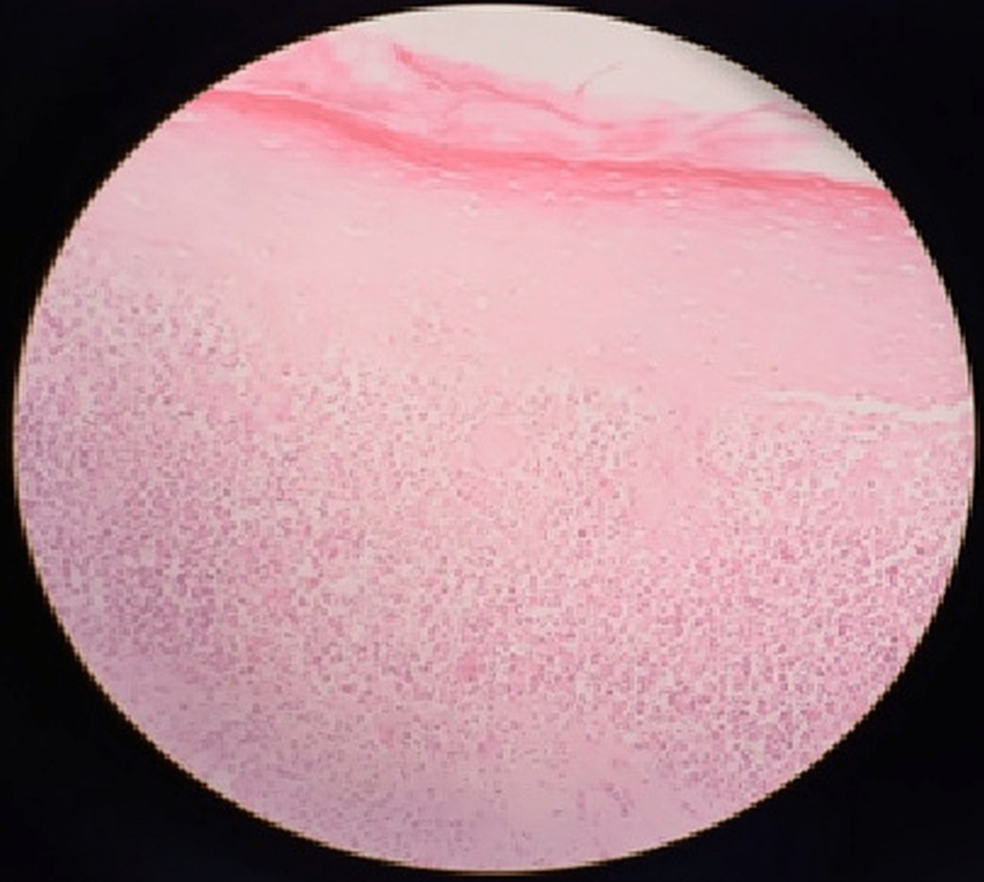
10× magnification of epithelial hyperkeratosis, acanthosis, saw-tooth rete pegs, and subepithelial lymphocytic infiltration

The entire study sample (100% reticular and erosive patterns) showed signs of hydropic degeneration in the layer of basal cells and the appearance of a clearly defined band-like region of inflammatory infiltration that is restricted to the superficial part of the connective tissue and is primarily made up of lymphocytes. One hundred and fifty-nine of the samples (64%), comprising 72% of reticular OLP and 52% of erosive forms, had hyperkeratosis. In 83 reticular cases (57%) and 19 erosive lesions (18%), acanthosis was found. Consequently, 41% of the study sample had acanthosis. One hundred and fifty cases (60%) were found to have saw-tooth ridges at the epithelial level, encompassing 90 (62%) cases of reticular lichen planus and 60 (57%) cases of erosive lichen planus. Neutrophils were not observed at the epithelial level in any of the reticular lesions, although they were seen in 60 specimens (57%) with erosive lichen planus. Figure 1 depicts 10× magnification of epithelial hyperkeratosis, acanthosis, saw-tooth rete pegs, and subepithelial lymphocytic infiltration.

**Figure 2:**
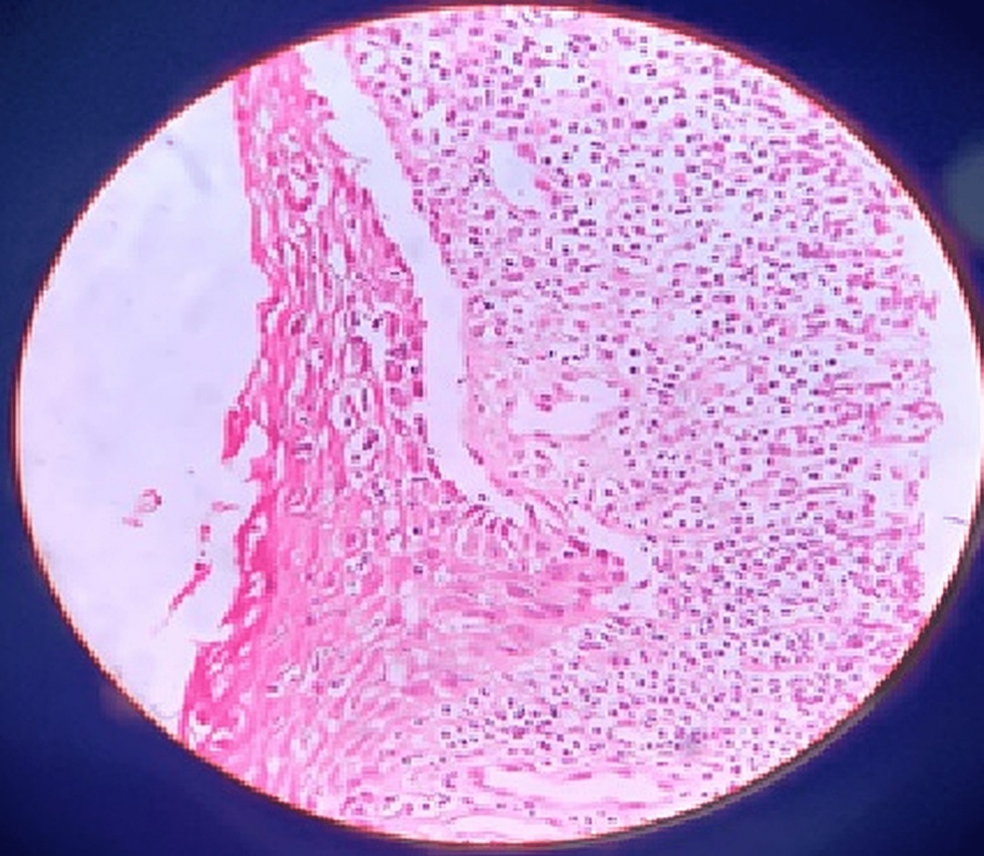
40× magnification of epithelial atrophy, hydropic degeneration of the basal layer saw-tooth rete pegs, subepithelial lymphocytic infiltration, and plasma cells in the connective tissue

Eighty-nine individuals (36%) had plasma cells found in the connective tissue; 45 cases (31%) had reticular OLP and 44 (42%) had erosive lesions. Figure 2 indicates 40× magnification of epithelial atrophy, hydropic degeneration of the basal layer, saw-tooth rete pegs, subepithelial lymphocytic infiltration, and plasma cells in the connective tissue.

**Figure 3:**
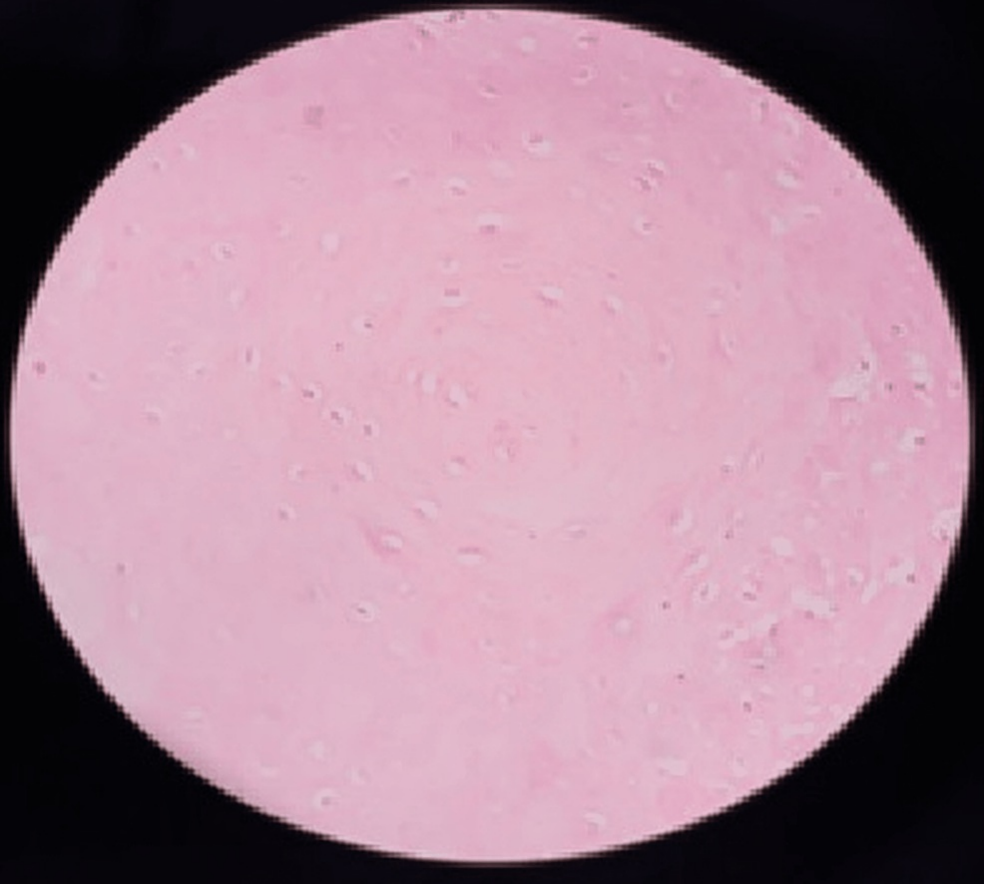
40× magnification of koilocytes showing vacuolated cells

Koilocytes were reported in 84 cases (34%), which included 35 (24%) reticular cases and 49 (47%) erosive lesions. Figure 3 represents 40× magnification of koilocytes showing vacuolated cells.

Histological features during the follow-up period

Repeat biopsies were performed on some of the lesions during the follow-up phase with the consent of the patient, particularly in the more critical cases such as worsening of the symptomatic form, changing from reticular to erosive form, or suspecting malignant transformation. It is crucial to note that 14 individuals (two with reticular form and 12 with erosive form) showed dysplasia, which was shown to be statistically highly significant. Moderate (n=2) to mild (n=12) dysplastic alterations were observed.

Clinical and histopathological relationship

The occurrence of plasma cells and saw-tooth rete pegs indicated statistically insignificant differences among the OLP histopathological features investigated. The histopathological features such as acanthosis, epithelial atrophy, hyperkeratosis, and neutrophil presence were shown to be statistically significant between the reticular and erosive forms (p<0.001).

## Discussion

The only clinical manifestations noted in the current investigation were reticular and erosive forms of lesions. It is not common to have plaque type and bullous type. This is not a common finding in the literature, and variations may be attributed to different clinical classifications of OLP, differing levels of interobserver variability, or adherence to stringent patient selection criteria [7]. The results of the present investigation were congruent with those of Torabi et al., who noted that the buccal mucosa is the most typical site for OLP, which can be ascribed to the epithelial thickness and the degree of keratinization [13]. According to De Sousa et al., the characteristic histological findings in OLP include lichenification of the basal layer, accompanied by a prominent layered lymphocytic infiltration explicitly beneath the epithelium; the existence of many eosinophilic colloid Civatte bodies along the epithelial-connective tissue interface; the lack of hyperplasic or, more frequently, saw-tooth interpapillary ridges; varying grades of orthokeratosis/parakeratosis; and differing thickness of spinous cellular layer [5].

Considering the histological findings, 100% of the sample population exhibited hydropic degeneration of the basal cell layer of the epithelium and a band-like subepithelial lymphocytic inflammatory infiltration, which is substantiated by other authors [3,11,14]. Sixty-four percent of the sample showed epithelial hyperkeratosis, which was more prevalent in the reticular type. Our findings on acanthosis (41%) agree with that reported in the previous reports [3,15]. However, the findings of Mravak-stipeti et al. documented that 77.6% of cases had acanthosis [16]. Another histological finding of OLP that was not compatible with the previous study [3] was the detection of saw-tooth ridges found in 60% of the sample. In the study sample, epithelial atrophy was not observed in any of the reticular cases, although being comparatively more common (40%) in the erosive forms. This might be the case because, as certain authors contend, the epithelium is thicker in the reticular forms and thinner in the erosive forms, rendering the erosive lesions more vulnerable to ulceration and atrophy [3,17]. In 57% of the erosive cases, neutrophils were found at the epithelial level, but none were found in the reticular pattern.

We identified band-like T lymphocytes predominated in the cellular infiltrate of the inflammatory process in 100% of the sample and plasma cells in 36% of the subepithelial connective tissue, which is in accordance with previous findings [3,7,15,16]. The cases evaluated in this study can be securely classified as OLP in their entirety as the diagnoses were made in accordance with the accepted OLP criteria. It was considered that one of the distinguishing characteristics of oral lichenoid reactions has been the presence of significant numbers of plasma cells. Before the study conducted by Fernández-González et al. [3], it was assumed that the clinical behavior of OLP was not associated with the existence of plasma cells, which raises concerns about the accuracy of the OLP diagnosis [15]. Both epithelial erosion and the deep extension of the inflammatory process were strongly associated with plasma cells in these patients. Plasma cells in the infiltration and profound diffusion around deeply embedded arteries in the connective tissue are not typical characteristics of OLP. The identification of plasma cells in OLP may be experienced due to reduced exacerbations and have a positive outcome to standard treatment with topical corticosteroids. Further prospective studies are required for this association to be evaluated [15]. Although there were no signs of exaggerated infection on either clinical or histological levels, subclinical infections cannot be entirely ruled out as the cause of the existence of the plasma cells [7]. Prior studies revealed that across observational periods of 0.5 to 20 years, OLP has been related to the risk of malignant transformation varying from 0.4% to 5% [14].

When dysplastic characteristics are present in the epithelium, there is a dispute about the pathogenesis of OLP [2]. Eisen [10] established that epithelial dysplasia was consistently seen in several repeat oral biopsies taken from a multitude of these patients. All patients with suspected OLP, with the possible exception of those who have characteristic, bilateral, white reticulated lesions, should have their diagnoses confirmed by biopsy because there are currently no standard, clearly defined clinical criteria for epithelial dysplasia [10,18]. Although the WHO views OLP as a condition that may progress to cancer, its precise likelihood of causing malignancy is the subject of considerable debate [19]. Lodi et al. asserted that the extremely persistent inflammatory process found in OLP causes the emergence of cell derangements that resemble those observed in epithelial dysplasia, but lack any malignant implications [20]. On the other hand, Mignogna et al. claim that chronic inflammatory responses provide a microenvironment in which cell viability, proliferation, and differentiation are altered, eventually leading to carcinogenesis, prompting such modifications to be regarded as potential indicators of malignant transformation [21].

The term "OLP with dysplasia" refers to lichen planus that has secondary dysplastic characteristics. Similar assumptions were made by Raj et al., who demonstrated that a provisional diagnosis of OLP with dysplasia could be confirmed by comparing it to one of the histological characteristics, such as degenerative changes in the basal cells, subepithelial inflammatory processes encompassing primarily lymphocytes, or the emergence of epithelial dysplasia of any degree or grade. A provisional diagnosis of OLP was established in the event of bilateral expression of the reticular pattern, with no history of amalgam restorations, the application of new medicaments or dentifrices, either with or without a history of usage of tobacco products, in addition to the aforementioned histopathological findings [22]. The definitive diagnosis of OLP depends heavily on the choice of the biopsy location. According to earlier research, reticular lesions were much more frequently histopathologically identified as OLP than erythematous and erosive lesions. As OLP develops through a sequence of exacerbations and remissions, there are certain occasions where the histopathological findings might not necessarily be diagnostic. However, a biopsy in almost any condition aids in determining if the lesions are caused by inflammation or have structural atypical alterations in the epithelium. It is worthwhile to take several biopsies because it is always probable that various ranges of disease processes could coexist. A biopsy of a cancer-prone or dubious location is more reliable if several biopsies are not feasible [9,23].

The development of epithelial dysplasia, in particular, has caused substantial debate regarding the potential malignant evolution of OLP. In routine clinical practice, epithelial dysplasia has proven to be the most effective marker for determining the likelihood of developing cancer in patients with the oral potentially malignant disorder (OPMD). Nevertheless, there is a significant deal of debate surrounding the widespread recognition of epithelial dysplasia as a concomitant histologic characteristic in OLP [18]. However, the results of this study concur with the observations of many experts and pathologists who have observed that certain cases of OLP with a solid clinical history eventually progress to epithelial dysplasia [18,24]. Although the present study documented two cases of mild dysplasia and 12 cases of moderate dysplasia, Odell et al. concluded that the presence/absence of dysplasia is undoubtedly more crucial than the grading. A severe/moderate dysplasia necessitates reconsidering additional samples, frequent follow-ups, and re-examination of other areas of the oral cavity as these regions may contain occult cancer [25].

Several oral lesions, notably OLP, leukoplakia, and oral epithelial dysplasia, harbored HPV-DNA, according to a systematic study by Syrjanen et al., with odds ratios of 4 to 5 in comparison to normal mucosa [26]. A subset of epithelial dysplasia was identified in the current investigation that shared the same characteristics as Woo et al. in terms of histological results and the occurrence of typical koilocytes [27]. Keratinocytes of the epithelium are frequently affected by HPV infection. Viral pathogens eventually enter cells in the basal cell layer through an eroded area of the epithelium, where they facilitate the production of regulatory proteins for the replication of DNA. Early viral genes cause basal cells to divide more rapidly, which causes hyperplasia in the superficial layers of the epithelium. Cells in the outermost surface layer go through a process known as koilocytosis, which involves nuclear degradation and perinuclear cytoplasmic vacuolation. Koilocytes are regarded as a hallmark of HPV infection based on histological evidence [28]. The rigorous criteria used in the current study for identifying koilocytes required that cells exhibit nuclear expansion, hyperchromasia, and distorted nuclear margins in addition to peri-nuclear halos. Besides this, similar to reports from other investigations, the study sample demonstrated substantial nuclear and cytoplasmic positivity for high-risk HPV subtypes [27].

Similar to the results of the current study, Singh et al. also found that oral submucous fibrosis, leukoplakia, OLP, and squamous cell carcinoma had higher incidences of koilocytes. Therefore, it is impossible to rule out the significance of HPV in the development of cancer [28]. If the cytology reveals the presence of the morphological cellular characteristics of koilocytosis, with the creation of a perinuclear halo and a thick cytoplasmic border, HPV infection could be anticipated. However, it is extremely likely to receive false-negative results while using cytological diagnostics. In addition, no other sensitive bioassay for detection exists because HPV is difficult to grow in an in vitro cell culture environment. The precise estimation and categorization of HPV genotypes are crucial for therapeutic applications, particularly in the detection and management of malignancies. The available molecular and genetic assays are expensive and complex [29]. Even while standard histology shows the presence of koilocytic cells, confirmation of these cells requires immunohistochemistry and more sophisticated methods like a polymerase chain reaction, electrophoresis assay, and DNA breakage detection fluorescence in situ hybridization. The literature has not yet progressed very far in exploring oral HPV infections. However, documentation of HPV infections is also becoming a key predictor for determining the prognostic value of head and neck cancer in recent times [8].

According to reports, the estimated prevalence of HPV associated with OPMD varies from 0% to 85%. The majority of research that examined HPV-DNA in OLP lesions used biopsy specimens, which confined their general usefulness solely for screening purposes. The quality and accuracy of DNA obtained from oral mucosa employing multiple collection and storage techniques remain a subject of debate [29]. The epithelium hypertrophy, conspicuous keratohyalin particles, acanthosis, and occasionally hyperkeratosis are epithelial proliferative symptoms caused by HPV [1]. Oral HPV-associated dysplasia can be identified by its distinctive histological characteristics, such as the presence of localized koilocytic alteration or vacuolation near the superficial layers, which are indicative of replicative HPV infection. This lesion seems to be uncommon, contributing to only an extremely small fraction of oral biopsy specimens with dysplasia. HPV-associated dysplasia has been identified as a distinct oral condition that can progress to cancer in a plethora of studies. Particularly, the presence of severe dysplasia with a heightened risk of malignant transformation is not always indicated by the involvement of the entire epithelial thickness. It is recommended that this condition be reported using the phrase "HPV-associated dysplasia" and that no grade be assigned until adequate follow-up records are obtained [25,27].

According to studies, erosive lichen planus patients have a higher likelihood to acquire malignant lesions. Odukoya et al. stated that epithelial atrophy has been reported to be a potential biomarker in malignant transformation, even if moderate-to-mild dysplasia might not always reflect premalignant potential [30]. OLP should indeed be monitored for prolonged periods of time, presumably for a lifetime, and be evaluated by biopsies with the primary purpose of determining the occurrence of epithelial dysplasia [18]. More investigation is required to analyze the significance of plasma cells and their presence in inflammatory infiltration for determining the prognosis of OLP. 

The study limitations are that the sample size is small and longitudinal evaluation was not done for the subjects as the OLP cases require a long-term follow-up. Various immunological tests have not been done in the cases for the lesions to be tested. 

## Conclusions

The results of the current study highlight the significance of taking into account clinicopathologic conclusions when establishing a definitive diagnosis. For comprehensive, accurate, and especially early diagnosis of OLP, the concordance of histopathological and clinical diagnoses becomes essential. More research studies are warranted to validate the trend of epithelial dysplasia in OLP associated with HPV and also to explore the course of the lesions that might be affected by this trait.
